# Intermediate Filaments at the Junction of Mechanotransduction, Migration, and Development

**DOI:** 10.3389/fcell.2017.00081

**Published:** 2017-09-14

**Authors:** Rucha Sanghvi-Shah, Gregory F. Weber

**Affiliations:** Department of Biological Sciences, Rutgers University-Newark Newark, NJ, United States

**Keywords:** intermediate filaments, development, migration, mechanotransduction, tension, keratin, vimentin

## Abstract

Mechanically induced signal transduction has an essential role in development. Cells actively transduce and respond to mechanical signals and their internal architecture must manage the associated forces while also being dynamically responsive. With unique assembly-disassembly dynamics and physical properties, cytoplasmic intermediate filaments play an important role in regulating cell shape and mechanical integrity. While this function has been recognized and appreciated for more than 30 years, continually emerging data also demonstrate important roles of intermediate filaments in cell signal transduction. In this review, with a particular focus on keratins and vimentin, the relationship between the physical state of intermediate filaments and their role in mechanotransduction signaling is illustrated through a survey of current literature. Association with adhesion receptors such as cadherins and integrins provides a critical interface through which intermediate filaments are exposed to forces from a cell's environment. As a consequence, these cytoskeletal networks are posttranslationally modified, remodeled and reorganized with direct impacts on local signal transduction events and cell migratory behaviors important to development. We propose that intermediate filaments provide an opportune platform for cells to both cope with mechanical forces and modulate signal transduction.

## Introduction

The last decade has brought a newfound recognition for the role of physical force on stimulating adhesion responses in cells, activating signal transduction pathways, and regulating cellular functions. These physical forces are recognized to be both externally and internally derived. Since the actomyosin machinery is seen as the primary means by which cells generate force, the focus of the research community thus far has largely been on the tripartite relationship between actin-myosin cytoskeleton, physical parameters such as force, and associated signal transduction pathways.

Interestingly, although it is well-accepted that the intermediate filaments provide mechanical integrity to cells, the relationship of intermediate filaments to mechanotransduction processes continues to be a new frontier ripe for exploration. Intermediate filaments remain the least well-understood of the three major cytoskeletal networks. Intermediate filament polymerization-depolymerization regulation, impact to cell signaling pathways and role in tension management within the cell have only just begun to be investigated. Findings to date provide good reason to anticipate an integral role for intermediate filaments in cellular processes where signal transduction and cellular mechanics converge.

In this review article, we provide a summary overview of what is currently known about cytoplasmic intermediate filaments and their regulation. The interplay of intermediate filaments with the cellular adhesive network is highlighted through multicellular behaviors such as migration. These cellular functions are put into physiological context by examining their contributions to embryonic development. We suggest that cytoplasmic intermediate filaments are load-bearing components within cells that both effect and are affected by cells' physical environments. That is, intermediate filaments are a centerpiece intracellular component of cell signaling events due to mechanical stimuli.

## Cytoplasmic intermediate filaments-general overview

Cytoplasmic intermediate filaments belong to a superfamily of highly conserved proteins (~65 genes; Hesse et al., 2001) unique to metazoan species (Herrmann and Strelkov, 2011). These members of the intermediate filament family likely originated through the divergence of the more ubiquitously expressed, yet highly conserved, nuclear intermediate filaments—the lamins (Dodemont and Riemer, 1990; Döring and Stick, 1990; Peter and Stick, 2015; Hering et al., 2016). The expression and assembly of these 10–12 nm filament forming cytoplasmic proteins is developmentally regulated in a cell-, tissue-, and context-dependent manner. Based upon their expression pattern, structure, and sequence identity, cytoplasmic intermediate filament proteins are classified into five different gene families (Table 1), with a sixth intermediate filament family comprised of lamins which reside in the nucleus (Type V). Type I and II consist of the keratins, which are intermediate filaments highly expressed by epithelia. Notably keratins are also expressed by several non-epithelial cell types and, of particular relevance to this review, are the earliest expressed intermediate filament types during embryogenesis (Franz et al., 1983; Lehtonen et al., 1983). In humans, keratins are encoded by 28 genes for Type I members and 26 genes for Type II members on only two loci (Hesse et al., 2001). While mice have a similar number of keratin genes, the number of keratin genes varies with species, with organisms lower on the phylogenic tree exhibiting fewer keratin genes. For instance, at the other end of the spectrum a single Type I-Type II keratin pair of genes exists in the sea squirt *Ciona intestinalis*, a difference indicative of the evolutionary expansion of keratins through gene duplication (Hesse et al., 2001; Karabinos et al., 2004). Type III intermediate filaments are intermediate filaments, such as vimentin and desmin, that can form homopolymers and also heteropolymers with other Type III intermediate filament proteins. Type IV intermediate filaments are expressed mostly by neurons and muscle and includes the various neurofilament subtypes, nestin and synemin. Type VI includes CP49/phakinin and filensin, which are lens-specific intermediate filaments. Although, CP49 can self-assemble *in vitro, in vivo* CP49 and filensin together form heteroligomeric filaments (Goulielmos et al., 1996). Here we will primarily focus on Type I-III cytoplasmic intermediate filaments, with special emphasis on keratin and vimentin, because of the emerging evidence for their influence over signal transduction, cellular function in a wide variety of cell types, and role in embryonic development.

**Table 1:** General classification of intermediate filament proteins.

**Intermediate filament type**	**General categorization**	**Intermediate filament protein members**
Type I	Acidic Keratins	Acidic Keratins (28 genes in humans)
Type II	Basic Keratins	Basic Keratins (26 genes in humans)
Type III	Homodimerizing intermediate filaments, some capability of heterodimerizing	Vimentin, Desmin, GFAP
Type IV	Intermediate filaments mainly expressed in neurons and muscle	Neurofilaments, Nestin, Synemin
Type V	Nuclear intermediate filaments	Lamins
Type VI	Lens-specific beaded intermediate filaments	Phakinin (CP49), Filensin

All cytoplasmic intermediate filament proteins share a common tripartite molecular structure. Intermediate filament monomers are dominated by a conserved central α-helical domain (~310 amino acids) flanked by highly variable non-α-helical head and tail domains. Variability in the amino terminal head domain and the carboxy terminal tail domains account for much of the diversity, specificity, and regulation of intermediate filaments. Meanwhile, the central domain is the primary dimerization region. The central rod domain is periodically interrupted by linker domains (L1 and L12) thus forming four helical subdomains (coil 1A, coil 1B, coil 2A, and coil 2B) capable of forming coiled-coils (Geisler and Weber, 1982; Nicolet et al., 2010; Chernyatina et al., 2012). These sub-helices are predominantly rich in heptad repeats (abcdefg)_n_ where a & d are the small apolar residues (Leu, Ile, Met, or Val). This amphipathic nature of the intermediate filament monomers allows them to readily form highly stable, parallel α-helical coiled-coil dimers without the assistance of any nucleating proteins *in vitro* (Quinlan et al., 1986).

Over the last few decades, several laboratories have elucidated the general mechanism for intermediate filament assembly *in vitro*. Cytoplasmic intermediate filament protein dimers laterally associate in an antiparallel fashion to form apolar tetramers. Once formed, tetramers further align to form unit length filaments (ULF's) that anneal longitudinally to yield long, flexible filaments, and subsequently undergo radial compaction to yield non-polar 10 nm filaments (Herrmann et al., 1996, 1999; Sokolova et al., 2006; Kirmse et al., 2007). Keratins spontaneously form obligate heterodimers from one Type I and one Type II intermediate filament protein (Steinert et al., 1976), whereas vimentin forms homopolymers (Steinert et al., 1981b). In both instances, nucleation and polymerization can occur without aid from co-factors or nucleoside triphosphates (Herrmann et al., 2004). Although, keratin heteropolymers can be formed from any combination of Type I acidic keratins and Type II basic keratins *in vitro*, assembly kinetics do show preferential pairing (Hatzfeld and Franke, 1985). Indeed, specific pairs of keratins are expressed *in vivo* (Franke et al., 1981) that parallel these assembly preferences. Such *in vitro* assembly studies of intermediate filaments, in addition to their remarkable insolubility in physiological buffers during *in vitro* experiments and resilient mechanical properties, led to the initial notion that intermediate filaments form stable networks in the cytoplasm.

While intermediate filament assemblages certainly have noteworthy physical properties, their assembly and disassembly are hardly static, unregulated, nor inconsequential to cell function. Contrary to *in vitro* assembly observations, *in vivo* pulse chase experiments suggest that intermediate filaments assemble from a soluble pool of tetrameric intermediate filament precursors/subunits (Blikstad and Lazarides, 1983; Soellner et al., 1985; Schwarz et al., 2015). Despite the tendency toward polymerization *in vitro*, in living cells intermediate filament proteins coexist both in filamentous form and as detergent-soluble filament precursors, of various varieties, including tetramers, ULFs, “particles,” and “squiggles”-short filamentous structures (Yoon et al., 1998, 2001; Schwarz et al., 2015). Intermediate filament precursors are most apparent in the peripheral region and protrusions of cells (Prahlad et al., 1998; Yoon et al., 1998, 2001; Helfand et al., 2002; Schwarz et al., 2015). As intermediate filament particles are transported, a subset of these are selected to elongate to short filaments otherwise known as squiggles and further assemble into mature filaments, finally incorporating into the network (Yoon et al., 1998; Windoffer et al., 2011; Schwarz et al., 2015). Cytoplasmic intermediate filaments establish complex networks that form a central cage like structure encapsulating the nucleus and further radiates toward the cell periphery (Franke et al., 1978a,b). Strikingly, when both the keratin and vimentin intermediate filaments are expressed in a cell type they display distinct spatial organization of intermediate filament arrays (Osborn et al., 1980). Typically keratin intermediate filaments are packaged into bundles called tonofibrils (Steinert et al., 1981a) and also form bifurcations (Nafeey et al., 2016). On the other hand, vimentin intermediate filaments are loosely arranged in a parallel or crisscross fashion forming a fine mesh network (Goldman et al., 1986). Polymerized filaments either become a part of the existing peripheral keratin intermediate filament network or get disassembled. Depolymerized precursors may be recycled in the cytoplasm and readied for the next cycle of assembly and disassembly or else undergo ubiquitin-mediated proteasomal degradation. Evidence points to a soluble pool of intermediate filament subunits as an important resource for the remodeling of intermediate filament networks. Keratin cycling uses disassembled soluble subunits for filament renewal, since inhibition of protein synthesis does not abolish filament formation nor subunit exchange (Kolsch et al., 2010). Similar cycling mechanisms are also seen for vimentin intermediate filaments, where vimentin precursors are translocated to the peripheral region of the spreading cells and display a stepwise formation of intermediate filaments (Prahlad et al., 1998).

In addition to dynamic assembly of intermediate filaments, established intermediate filament networks are subject to marked remodeling. Live imaging studies highlight the dynamic and motile properties of the intermediate filament networks and clearly show that vimentin and keratin fibrils exhibit undulations while constantly changing their configurations, appearing to collapse, extend, and translocate over relatively short time intervals (Ho et al., 1998; Yoon et al., 1998; Windoffer and Leube, 1999). At the subunit level, the dynamic exchange of intermediate filament dimers is ever more complex. Predictably, severing and end-to-end annealing are major mechanisms for elongation and refurbishment of filaments (Prahlad et al., 1998; Wöll et al., 2005; Winheim et al., 2011; Hookway et al., 2015). Breaking with typical models of filament polymerization however, data show that intermediate filament subunits can also be added and removed from the entire length of the preformed filament as a mechanism for intermediate filament turnover (Ngai et al., 1990; Coleman and Lazarides, 1992; Vikstrom et al., 1992). Microinjection of soluble biotinylated keratin and vimentin (Vikstrom et al., 1989; Miller et al., 1991) and rhodamine-tagged vimentin followed by Fluorescence Recovery After Photobleaching (FRAP) analyses revealed that these subunits can rapidly incorporate in a well-established network (Vikstrom et al., 1992). Exchange of intermediate filament subunits is non-polar and occurs along the entire length of the intermediate filament. Nevertheless, subunit swapping is highly dependent on the availability of a soluble pool. Collectively, this suggests that established intermediate filaments exhibit unique dynamic properties that allow for turnover of subunits, along with severing and end-to end annealing to maintain length and flexibility of the preformed filaments without compromising their structural integrity. Factors regulating and facilitating processes such as severing, reannealing and subunit exchange are largely unknown and the underlying molecular mechanisms demand further attention given the disparate assembly dynamics *in vitro* compared to *in vivo*. As with their actin and microtubule counterparts, we speculate that a host of intermediate filament binding proteins that modulate polymerization are waiting to be elucidated.

Despite the ability of keratin and vimentin to polymerize so readily, cells clearly have regulatory controls that determine when and where intermediate filaments will form. A variety of post-translation modifications (PTMs) of intermediate filaments such as phosphorylation, ubiquitylation, acetylation, and sumoylation are emerging as crucial controllers of intermediate filament dynamics, stability, and function (Table 2; for review see Snider and Omary, 2014). Phosphorylation status of cytoplasmic intermediate filaments is modulated by various kinases and phosphatases (Omary et al., 2006) depending upon the intermediate filament protein involved, cell and tissue type and specific physiological condition. Such phosphorylation/dephosphorylation events in general affect their conformation, solubility, filament organization, and interaction with other signaling molecules consequently translating into various cellular functions. Site-specific serine/threonine phosphorylation of the head and tail region of intermediate filaments facilitate their structural reorganization mainly by promoting intermediate filament solubility. Conversely, tyrosine phosphorylation of the rod domain (Tyr 267) of K8 facilitates their proper filament formation and renders them insoluble (Snider et al., 2013b). Different PTMs on intermediate filaments can serve as docking sites for protein complexes and allow for altered assembly dynamics or rearrangements of the network. For example, the chaperone protein Hsp27 interacts with K8 in a phosphorylation dependent manner (Kayser et al., 2013). Hsp27 manages keratin inter-filament interactions by inhibiting extensive bundling of filaments (Kayser et al., 2013), in essence by acting as a steric “spacer.” Such phosphorylation events not only correlate with, but have a functional role in cellular events such as mitosis, cell migration, cell growth, and stress-mediated responses. Spatiotemporal localization of intermediate filaments is phosphorylation dependent. Different forms of intermediate filament proteins (e.g., non-filamentous particles vs. filaments) reside in different subcellular compartments. During distinct cellular processes such as mitosis, site-specific hyperphosphorylation of cytoplasmic intermediate filaments, as determined by phospho-specific antibody labeling, affect their organization and distribution (Chou et al., 1991; Toivola et al., 2002). Epitope-specific phosphorylation can also segregate intermediate filaments to different compartments within a cell. In pancreatic acinar cells, K18 phosphorylated on Ser33 is necessary for specifically basal filament organization while the non-phosphorylated K18 localizes to the apical domain by default (Ku et al., 2002). Moreover, similar site-specific intermediate filament phosphorylation can be limited to specific cells within a tissue. For example, K20 Ser13 phosphorylation occurs specifically in small intestine goblet cells but not in other K20 expressing enterocytes (Zhou et al., 2006).

**Table 2:** Post-translational modifications of cytoplasmic intermediate filaments.

**Post-translational modification**	**General effect on intermediate filament**	**References**
Ser/Thr phosphorylation	Increases intermediate filament solubility	Omary et al., 2006; Snider and Omary, 2014
Tyr phosphorylation	Promotes keratin insolubility	Snider et al., 2013b
Ser phosphorylation	Induces compartmentalization of intermediate filaments	Chou et al., 1991; Ku et al., 2002; Toivola et al., 2002; Zhou et al., 2006
Sumoylation	Alters filament dynamics	Snider et al., 2011
Acetylation	Promotes formation of dense perinuclear network	Snider et al., 2013a
Glycosylation	Protects against stress and injury	Ku et al., 2010
Ubiquitylation	Regulates intermediate filament degradation and turnover	Ku and Omary, 2000

Phosphorylation of intermediate filaments promotes other PTMs, such as sumoylation of keratins. Both keratins and vimentin are extensively modified by SUMO2 and SUMO3 *in vitro*, at multiple conserved rod domain sites. Monosumoylation of keratin increases protein solubility whereas hypersumoylation decreases it (Snider et al., 2011). Thus, sumoylation similar to phosphorylation is crucial for regulating solubility of cytoplasmic intermediate filaments. Similarly, acetylation of the conserved Lys residue (Lys 207) in the rod domain of K8 also decreases K8 solubility. Acetylation thus promotes the formation of a dense perinuclear intermediate filament network formation altering the mechanical properties of filaments (Snider et al., 2013a). A plausible reciprocal relationship exists between glycosylation and phosphorylation PTMs of intermediate filaments (Ku et al., 1996). Glycosylation of K18 (on Ser30/31/49 in the head domain) behaves as a cytoprotective PTM during stress and injury (Ku et al., 2010), whereas ubiquitylation is frequently detected in the context of hyperphosphorylated intermediate filaments. Ubiquitination of intermediate filament proteins promotes intermediate filament turnover; for example, increased K8 and K18 synthesis and phosphorylation predisposes these proteins to ubiquitination-dependent degradation (Ku and Omary, 2000). Thus, different PTMs by themselves or in combination not only alter the intermediate filament organization but also influence the recruitment of intermediate filament accessory proteins, in a context dependent fashion which subsequently regulate various cellular processes.

## Intermediate filaments and the establishment of a cellular architectural framework

Throughout development, cells both exert and are subject to an array of forces. These physical interactions are initiated not only by the extra-organismal environment, but also by neighboring cells and extracellular matrix. To maintain the integrity of multicellular tissues, cells must (1) avoid rupturing due to mechanical strain and (2) remain adherent to one another. Intermediate filaments have unique features that not only distinguish them from the other two cytoskeletal elements, actin and microtubules, but also make them major contributors in providing mechanical resistance to the cells (Table 3). The persistence length of intermediate filaments is much shorter than both actin microfilaments and microtubules, thus classifying them as flexible polymers (Gittes et al., 1993; Mücke et al., 2004; Schopferer et al., 2009; Lichtenstern et al., 2012; Nöding et al., 2014; Pawelzyk et al., 2014). Cytoplasmic filaments along with being flexible and elastic are also highly extensible and can be stretched ~2.8-fold without rupturing (Kreplak et al., 2005). Microfilaments and microtubules are more fragile and tend to rupture at strains <50% (Janmey et al., 1991). Furthermore, intermediate filaments exhibit strain-induced strengthening without catastrophic failure, making them very suitable as intracellular load bearing springs (Ackbarow et al., 2009; Pawelzyk et al., 2014). Both *in vitro* and *in vivo* analyses corroborate this conceptual model of intermediate filaments as important contributors to cells' elasticity and tensile strength (Janmey et al., 1991; Ma et al., 1999; Fudge et al., 2008; Nolting et al., 2015). The dominant function of intermediate filaments in defining cell stiffness is emphasized in keratinocytes devoid of the entire keratin cytoskeleton (Ramms et al., 2013; Seltmann et al., 2013a). Indirect perturbation of cytoplasmic intermediate filaments likewise has detrimental effects on cell stiffness. Cells exposed to lipids such as sphingosylphosphorylcholine (SPC), induce perinuclear reorganization of keratins through site-specific phosphorylation, leading to a marked decrease in the elastic modulus (Beil et al., 2003). Studies using keratin mutants that either mimic or abrogate phosphorylation of keratins at specific sites further underscore the importance of phosphorylation on the mechanical properties of intermediate filaments (Fois et al., 2013; Homberg et al., 2015). Although tensile strength is most often attributed to the keratin filaments present in epithelial cells, vimentin also contributes to structural integrity, such that cell stiffness is reduced in vimentin depleted or disrupted cells (Wang and Stamenović, 2000; Gladilin et al., 2014; Sharma et al., 2017) and stiffness is increased in cells overexpressing vimentin (Liu et al., 2015). Vimentin further protects fibroblasts against compressive strain (Mendez et al., 2014).

**Table 3:** Comparison of the mechanical properties of cytoskeletal elements.

	**Keratin intermediate filaments**	**Vimentin intermediate filaments**	**Actin filaments**	**Microtubules**
	**Lp<L (flexible)**		**Lp_~_L (semi flexible)**	**Lp>>L (rigid)**
Persistence length (Lp)	0.3–0.65 μm Lichtenstern et al., 2012; Pawelzyk et al., 2014	0.4-2 μm Mücke et al., 2004; Schopferer et al., 2009; Lin et al., 2010; Nöding et al., 2014	18 μm Gittes et al., 1993	1,000–5,000 μm Gittes et al., 1993
Contour length	10–20 μm	10–20 μm	≤1 μm	5–15 μm
Extensibility	~280^+^% Kreplak et al., 2005	~300% Qin et al., 2009	~20% Janmey et al., 1991 ~200% (native stress fibers) Labouesse et al., 2016	~50% Janmey et al., 1991

Along with maintaining the general mechanical integrity of the cytoplasmic volume, cytoplasmic intermediate filaments are also vital determinants of intracellular organelle organization. Vimentin plays a critical role in influencing actin and Rac1 driven (Dupin et al., 2011; Matveeva et al., 2015) localization of cytoplasmic organelles such as endoplasmic reticulum, Golgi complex, nucleus, and mitochondria (Gao and Sztul, 2001; Nekrasova et al., 2011; Guo et al., 2013). In *Xenopus laevis*, vimentin intermediate filaments form a cage around melanophores and are involved in their transport and localization at distinct sites within the cells (Chang et al., 2009). Vimentin intermediate filaments are also involved in endoplasm spreading (Lynch et al., 2013). Nuclear position and shape have emerged as important downstream outcomes of mechanical stimuli. Changes in nuclear position relative to other organelles can determine cell polarity, and modulation of nuclear shape influences gene expression and stability. Cytoplasmic intermediate filaments physically link to the nuclear envelope via plectin (an intermediate filament-interacting protein) and SUN/nesprin complexes, also known as Linker of Nucleoskeleton and Cytoskeleton (LINC) complexes (Wilhelmsen et al., 2005; Ketema et al., 2007; Burgstaller et al., 2010). Targeted deletion of nesprin-3 or expression of dominant negative nesprin alters perinuclear intermediate filament organization, cell polarization, and migration (Lombardi et al., 2011; Morgan et al., 2011; Postel et al., 2011). Plectin knockout or plectin mutations related to severe skin blistering disease (epidermolysis bullosa simplex) impair perinuclear keratin architecture, but not the linkages to the nucleus, nonetheless leading to misshapen nuclei and abnormal nuclear deformability (Almeida et al., 2015).

At the cell periphery, adhesions to neighboring cells and the surrounding extracellular matrix provide the interface with which cells interact. Intermediate filaments are most commonly known to be anchored to adjacent cells by desmosomes and to the extracellular matrix by hemidesmosomes. The classic view is that intermediate filaments simply provide internal scaffolding attachments to desmosome and hemidesmosome complexes, adhesive junctions that convey relatively long term associations between a cell and its environment. However, the association of keratin intermediate filaments with desmosomes and hemidesmosomes is also mechanistically supportive of the molecular adhesive complex. That is, keratin filaments can promote the formation and maintenance of desmosomes and hemidesmosomes (Kröger et al., 2013; Seltmann et al., 2015; Loschke et al., 2016). Reciprocally, desmosomal adhesions act as organizing centers for *de novo* keratin network formation in native state tissues (Jackson et al., 1980; Schwarz et al., 2015).

In addition to this classical view of intermediate filaments associating with hemidesmosomes and desmosomes, intermediate filaments interact with cell adhesions often inaccurately believed to be exclusively actin-linked, including junctions mediated by classical cadherins (Kim et al., 2005; Leonard et al., 2008; Weber et al., 2012) and focal adhesions (Tsuruta and Jones, 2003; Windoffer et al., 2006). Association of the intermediate filaments with these otherwise actin-linked adhesions is bolstered in response to physical forces applied to these adhesions (Tsuruta and Jones, 2003; Weber et al., 2012). As with desmosomes and hemidesmosomes, intermediate filaments modify the stability of actin-linked focal adhesions and classical cadherin adhesions. In endothelial cells, vimentin regulates the size and adhesive strength of focal adhesions (Tsuruta and Jones, 2003; Bhattacharya et al., 2009). Vimentin is also implicated in the regulation of vesicular transport of integrin toward the cell membrane (Ivaska et al., 2005).

Intermediate filaments are physically linked to these various adhesion complexes. Vimentin intermediate filaments interact with integrins either directly with binding to β3 integrin tail (Kim et al., 2016) or indirectly via linker proteins including plectin (Bhattacharya et al., 2009; Burgstaller et al., 2010; Bouameur et al., 2014) and BPAG (bullous pemphigoid antigens 1 and 2) by forming dynamic linkages with plakin repeat domains (Fogl et al., 2016). In *X. laevis* mesendoderm (also known as anterior head mesoderm) cells, plakoglobin acts as recruitment signal for keratin intermediate filament association with cadherins (Weber et al., 2012). Similarly, in endothelial cells p120 catenin recruits vimentin intermediate filaments to cadherins (Kim et al., 2005). Both vimentin and keratin precursor assembly show dependence on focal adhesions as recruitment sites for motile precursors (Burgstaller et al., 2010). Tethering of adhesion complexes to intermediate filaments presents an ideal circumstance wherein intermediate filaments can serve as mediators of both tension and the coincident signaling that we now know occurs as a function of these adhesions.

Although intermediate filaments, microtubules and actin cytoskeletal networks are often viewed as three separate entities, these filamentous arrays cooperatively interact in more ways than not. Actin filaments and microtubules both have impacts on intermediate filament organization through multiple direct, indirect and steric interactions. Bidirectional motility of both mature filaments and their non-filamentous precursors of intermediate filaments can occur on either microtubules or actin microfilaments (Prahlad et al., 1998; Helfand et al., 2002; Liovic et al., 2003; Wöll et al., 2005; Kölsch et al., 2009; Hookway et al., 2015). The filament network used to transport intermediate filament precursors is entirely dependent on context. Neither keratin nor vimentin seems to be exclusively limited to microtubules or actin. Motility of intermediate filament precursors can be both fast and slow, in retrograde and anterograde. Although the mechanism of transport seems to be well-defined in some cases (e.g., entirely dependent on actin or microtubules), defining trends have yet to emerge that reliably predict a mechanism of transport for intermediate filaments. Actin filaments are essential to retrograde motility of keratin precursors in some epithelial cells (Kölsch et al., 2009), yet actin cytoskeleton can restrict microtubule-dependent vimentin precursor movement, establishing a complex three-way cytoskeletal communication (Robert et al., 2014). Perturbing either microtubules, microfilaments, or their associated molecular motors can lead to intermediate filament collapse (Knapp et al., 1983; Wöll et al., 2005). Absence of vimentin intermediate filaments alters the microtubule network orientation, thus suggesting a function of vimentin in organizing the cytoskeletal architecture necessary for cell polarity (Shabbir et al., 2014; Liu et al., 2015). In addition, vimentin filaments, but not “non-filamentous” vimentin negatively regulate actin stress fiber assembly and contractility (Jiu et al., 2017). *In vitro* and *in vivo* studies have highlighted direct interactions between the tail domain of vimentin and actin (Cary et al., 1994; Esue et al., 2006). Furthermore, numerous cytoskeletal linkers have been identified that allow for indirect interaction among the cytoskeletal polymers. These proteins include plectin (Svitkina et al., 1996; Osmanagic-Myers et al., 2015), myosin (Kölsch et al., 2009; Robert et al., 2014), fimbrin (Correia et al., 1999), filamin A (Kim et al., 2010), kinesin (Prahlad et al., 1998; Kreitzer et al., 1999), adenomatous polyposis coli (APC; Sakamoto et al., 2013), dynein, and dynactin (Helfand et al., 2002). Along with distinct cytoskeletal entities interacting with one another, vimentin and keratin intermediate filament networks have been observed to interact at the helical 2B domain of vimentin, and mutations in this region negatively impact collective cell migration (Velez-delValle et al., 2016).

**Figure 1:**
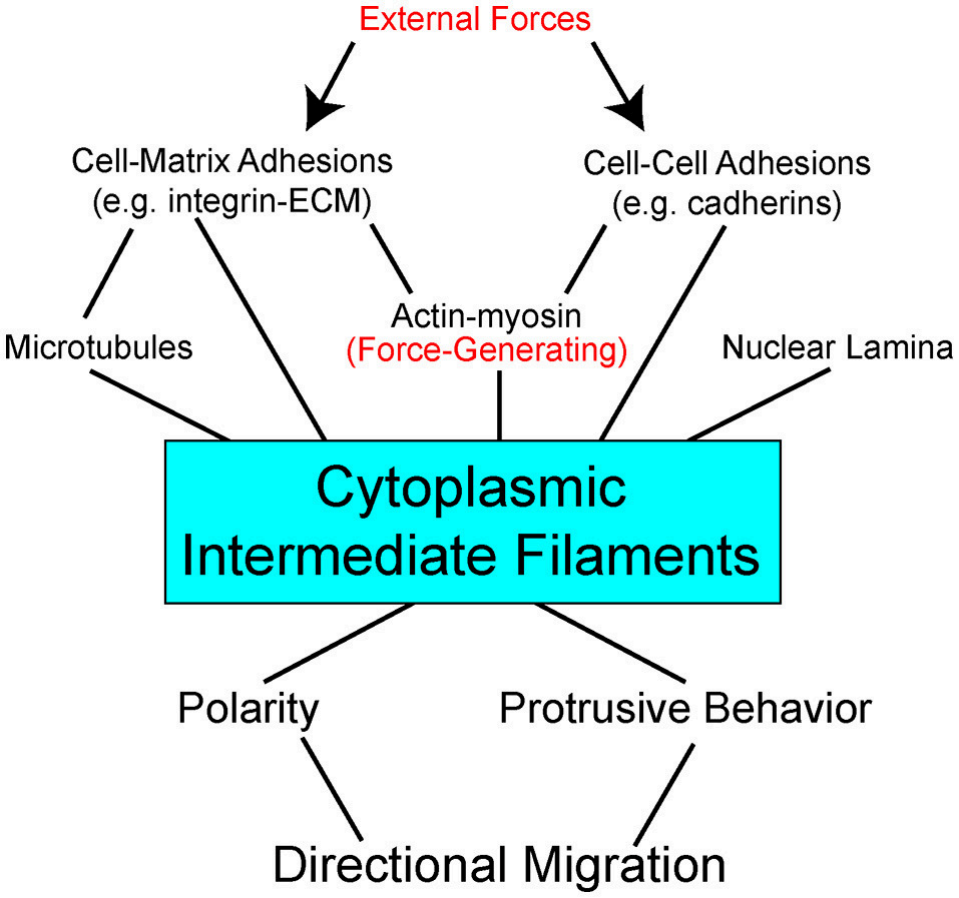
Interdependent network model of cytoplasmic intermediate filaments as a centerpiece between mechanical stimuli and directional cell migration. External forces act on (arrows) adhesion molecules on the cell surface to impact a complex network of bidirectional interactions within the cell (lines). Adhesions are linked to the three major cytoskeletal networks. Of these, actin with its myosin motors is the primary force-generating apparatus. Intermediate filaments can be pre-stressed by actomyosin generated tension. Intermediate filaments also act to resist strains imposed on the cell. Through modulation of cell signaling pathways, direct and indirect, intermediate filaments effect cell polarity and protrusive behavior. Stabilization of distinct subcellular locales promotes persistent directional migration.

Some data suggest specific roles for each of the cytoskeletal networks in how a cell normally manages different physical forces and stresses. And yet when systems are disrupted, cells often find ways to compensate using the available “protein toolkit.” Mechanical probing of fibroblasts shows that actin contributes to cortical stiffness, whereas vimentin dominates cytoplasmic stiffness (Guo et al., 2013). Disruption of vimentin intermediate filaments mandates that cells find other ways of dealing with imposed forces. In some cases, cells compensate to accommodate self-generated forces by increasing actin stress fibers and myosin activity to facilitate ECM substrate traction while exhibiting disruption of cell-cell adherens junctions (Osmanagic-Myers et al., 2015; Jiu et al., 2017). In response to increased externally-derived physical strain and mechanosensing of these forces, keratins can promote stress fiber formation and cell stiffness by activation of ROCK signaling pathway (Bordeleau et al., 2012). These data illustrate great versatility in how cells use the cytoskeletal networks available to facilitate adhesion, cohesion, and balance intracellular tension and externally-derived stresses. In the context of the complex multicellular animal, keratin, and vimentin establish an important scaffolding framework inside the cell. Cytoplasmic intermediate filaments enable the cells to resist deformation, localize organelles, change shape, and are integrally coupled to adhesion complexes (Figure 1).

## Intermediate filaments and modulation of signal transduction pathways

Intriguingly, a wide range of findings suggest that functions of intermediate filaments extend well beyond the mechanical and structural to direct participation in signal transduction. Interactions between cytoplasmic intermediate filaments and other cellular proteins initiate signaling cascades that regulate responses to process such as growth, migration, and apoptosis—all cellular processes fundamental to development and embryogenesis.

Cytoplasmic intermediate filaments differentially regulate cellular adhesions through effecting signaling pathways. Vimentin intermediate filaments promote the formation, maturation, and adhesive strength of maturing focal adhesions (Tsuruta and Jones, 2003; Bhattacharya et al., 2009; Burgstaller et al., 2010; Lynch et al., 2013; Liu et al., 2015). Vimentin regulates VAV2, a Rac1 GEF, phosphorylation, and localizes phosphorylated VAV2 to focal adhesions to promote Rac1-mediated focal adhesion kinase (FAK) stabilization, which further stabilizes focal adhesions (Havel et al., 2015). PKCε-mediated phosphorylation of vimentin governs efficient β1-integrin recycling and motility (Ivaska et al., 2005), whereas Cdc2-mediated vimentin phosphorylation promotes β1-integrin activation, leading to FAK phosphorylation (Chang et al., 2012). Likewise, keratins dynamically regulate focal adhesions through integrin/FAK-dependent signaling mediated either via PKCδ or Akt signaling (Bordeleau et al., 2010; Sankar et al., 2013). Reciprocally, during collective cell migration FAK is required for cadherin-dependent keratin intermediate filament organization (Bjerke et al., 2014). Keratins display isotype-specific signaling functions and unique gene expression patterns of keratins can control stability and dynamics of adhesion complexes. For example, K5/K14 inhibit PKCα-mediated phosphorylation of desmoplakin via RACK1 and thus promote stability and maintenance of desmosomal junctions (Kröger et al., 2013). K6 similarly suppresses cell motility by sequestering Src from being activated and targeting focal adhesions (Rotty and Coulombe, 2012). In contrast, induction of K6/K17 expression produces PKCα-mediated desmosome disassembly (Loschke et al., 2016). Intermediate filaments are tightly coupled with signaling pathways to modulate cellular adhesions.

An ever increasing number of studies implicate intermediate filaments as signaling platforms and as scaffolds for signaling proteins. In fibroblasts, intermediate filament-associated plectin molecules sequester RACK1 on intermediate filaments to modulate PKCδ function (Osmanagic-Myers and Wiche, 2004). Similarly, in keratinocytes, plectin-mediated concentration of RACK1 further regulates ERK1/2 pathway (Osmanagic-Myers et al., 2006). In HER2 positive tumors, a positive feedback loop exists which induces K19 expression via HER2/ERK and further stabilizes HER2 on cell membrane by Akt mediated phosphorylation of K19 on Ser35 (Ju et al., 2015). Keratin 19 is also involved in shuttling β-catenin/Rac1 complex into the nucleus and thus modulating NOTCH signaling pathway in breast cancers (Saha et al., 2017). Collectively, these results support numerous scaffolding roles for keratin intermediate filaments. Vimentin intermediate filaments also function as scaffolds for ERK activation (Kumar et al., 2007). An interesting positive feedback loop exists between vimentin and ROCK2 activation. Activation of ROCK2 causes intermediate filament collapse with simultaneous release of inactive ROCK2. The released ROCK2 is translocated to the periphery where it gets activated again and acts on phosphorylated intermediate filaments (Sin et al., 1998). The vimentin interacting protein Raf-1 associated kinase in concert with other vimentin kinases induce extensive vimentin reorganization (Janosch et al., 2000). It is well-known that intermediate filament architecture is reorganized in response to cell migration stimuli. α-Catulin, a scaffold for the ROCK signalosome (Park et al., 2002), co-localizes with vimentin intermediate filaments and contributes to vascular endothelial cell migration (Bear et al., 2016). In addition, vimentin filaments are also essential for VASP localization and phosphorylation by cGMP dependent kinase in endothelial cells (Lund et al., 2010). Many studies have emphasized the contributions of polymerized intermediate filaments, but a novel role of soluble vimentin precursors has been proposed that is not necessarily related to biogenesis of intermediate filament network. Here, soluble vimentin molecules bind to importin-β and phosphorylated ERK and thus enable their retrograde transport in sensory axons (Perlson et al., 2005, 2006). Polymerized intermediate filaments can function as scaffolds while even intermediate filament precursors can play a role of adaptors to transport signaling molecules.

Cytoplasmic intermediate filaments can also serve as phosphate “sponges or sinks,” with broad implications to all signal transduction events. In particular, their head and tail domain have been proposed to buffer excess kinase activity. Such hyperphosphorylation of intermediate filaments can be detrimental or advantageous for the cell depending on the biological context (Lai et al., 1993; Ku and Omary, 2006). Phosphorylated cytoplasmic intermediate filaments can also act as sequestering reservoirs to accommodate stress response or physiological processes. Both phospho-vimentin and keratin provide binding affinity to sequester proteins like 14-3-3 and therefore limit their availability to other target proteins in order to regulate processes like mitosis and signal transduction (Tzivion et al., 2000; Kim et al., 2006; Margolis et al., 2006). Cell proliferation and size are closely coupled to the binding of adaptor proteins and kinases to cytoplasmic intermediate filaments serving as either molecular scaffolds or sequestration sinks. Perhaps the best example of phosphorylated intermediate filaments operating as docking sites for proteins is provided by members of the 14-3-3 protein family. Keratins and vimentin orchestrate the local interaction of 14-3-3 proteins with their multiple binding partners. 14-3-3 proteins bind keratin 18 (K18) at Ser33 in a cell-cycle and phosphorylation-dependent fashion (Ku et al., 1998) and trigger keratin filament solubilization during hepatocyte mitotic progression (Ku et al., 2002). K18 and 14-3-3 interaction is closely coupled to the association of 14-3-3 proteins with a host of phosphorylated signaling molecules that are involved in mitotic progression, such as Raf1 kinase and Akt (Deng et al., 2012). In the case of Raf, K8/K18 filaments regulate cell signaling via the known K18 and 14-3-3 complex and recruitment of Raf1 kinase by 14-3-3 (Ku et al., 2004). Similarly, phosphorylated vimentin also provides a binding sink for 14-3-3 adaptor proteins (Tzivion et al., 2000). Physical interaction of K10 with Akt and atypical PKCζ inhibits intracellular translocation of these kinases, thus modulating PI-3 kinase signal transduction pathway and enabling K10 to function as a negative modulator cell cycle progression (Paramio et al., 2001). K17/14-3-3 complex has the ability to stimulate Akt/mTOR signaling and influence epithelial cell growth and size by regulating protein synthesis (Kim et al., 2006). Thus, phosphorylation of intermediate filaments has broad impacts to both intermediate filament polymerization status as well as modulation of cell signaling pathways.

## Role for intermediate filaments in mechanotransduction

Cellular mechanotransduction is an integration of multiple mechanical cues derived from sensing, transmission of force, and transduction into a biochemical response. There is extensive evidence that cell-cell, cell-ECM, and flow forces are actively sensed in different cellular contexts by the junctional protein complexes (Riveline et al., 2001; Weber et al., 2012; Conway et al., 2013). Different mechanical forces alter the structure, assembly, adhesive strength, function, and signaling of these adhesive complexes, which in turn has consequences to the cytoskeleton. Cytoplasmic intermediate filaments behave as an elastic and conductive network to transmit force and propagate mechanical stimuli within and between cells via adhesion complexes. As we detailed above, cytoplasmic intermediate filaments emerge as modulators of specific signal transduction pathways in a variety of biological contexts. Abundant availability, overall cytoplasmic presence and subcellular reorganization dependent on cellular context, allows the cytoplasmic intermediate filaments to partake in various signaling pathways in a multitude of ways. Such a view presents cytoplasmic intermediate filaments to be apt to transduce mechanical stimuli during development while integrating an ever changing physical environment with cell signaling (Figure 1).

Fluid flow shear stress plays important roles in the developing vasculature system. Perhaps more surprisingly fluid flow shear stress is also an important mechanical stimulus in tissues not often intuitively associated with exposure to fluid flow shear stresses, such as bone and glandular epithelia. Fluid flow shear stress studies have shed some light on the role of intermediate filaments in mechanotransduction pathways. Cytoplasmic intermediate filaments alter their network organization most likely by mechanisms such as conformational change, changes in assembly, PTMs and others. Mechanical forces such as shear stress can induce rapid reorganization of vimentin and keratin intermediate filament networks in various cell types, suggesting a role in spatial redistribution of intracellular force (Helmke et al., 2000, 2001; Yoon et al., 2001; Sivaramakrishnan et al., 2008). Shear stress increases the keratin intermediate filament network stiffness in the peripheral region of the cytoplasm (Sivaramakrishnan et al., 2008). Shearing also dramatically transforms the keratin intermediate filaments into more “wavy” tonofibril bundles, a process that is promoted by K8 and K18 phosphorylation on serine residues 73 and 33, respectively (Flitney et al., 2009; Sivaramakrishnan et al., 2009). This phosphorylation dependent reorganization of intermediate filaments is regulated by a variety of protein kinases including PKCδ and PKCζ (Ridge et al., 2005; Sivaramakrishnan et al., 2009). Phosphorylation of keratins in the regulatory head domain (K18 pSer33) recruits binding of 14-3-3, which allow for dynamic exchange and remodeling of the network (Sivaramakrishnan et al., 2009). In order to control against hyperphosphorylation induced disruption, keratin intermediate filaments recruit epiplakin, which perhaps serves as a chaperone during filament reorganization (Spazierer et al., 2008). In addition to the local deformation of the intermediate filaments, there is increased association of vimentin intermediate filaments with β3-integrin focal contacts, further stabilizing cell-matrix adhesions (Tsuruta and Jones, 2003). Similarly, in response to shear stress, endothelial cells trigger a transition from cell-cell adhesion loading on VE-cadherin to interaction of PECAM (Platelet endothelial cell adhesion molecule-1) with vimentin to stabilize cell-cell junctions (Conway et al., 2013). In this manner, mechanical loads may be transferred from one cytoskeletal network to another. Indeed keratin intermediate filaments exhibit less motion when actin-myosin II rigidity is increased, likely a consequence of stress generated by actomyosin being transmitted to pre-stress the keratin intermediate filament network (Nolting and Koster, 2013).

Experimentally introduced physical forces, induced by optical tweezers and fibronectin beads on epithelial cells, promote the modulation of both the K8/K18 intermediate filaments and the actin network through Rho-ROCK pathway (Bordeleau et al., 2012). Tensile forces reinforce stress fibers by joint coordination between Solo protein a RhoA GEF and K8/K18 intermediate filament network (Fujiwara et al., 2016). Actin stress fiber assembly and contractility are likewise modulated by vimentin filament dependent regulation of RhoA and GEF-H1 (RhoA GEF protein; Jiu et al., 2017). Decoupling the intermediate filaments from the mechanotransduction pathway has revealed hitherto unrecognized roles of intermediate filaments in this process. For instance, cells with inhibited vimentin expression display reduced mechanical resistance to the effects of flow (Tsuruta and Jones, 2003). Likewise mutant keratin intermediate filament network is unable to withstand mechanical stress (Ma et al., 2001), with marked reorganization of the filaments into discrete aggregates (Russell et al., 2004). In the absence of vimentin intermediate filaments or their displaced anchorage due to loss of plectin, cells display compromised activation of FAK and its downstream targets Src, ERK1/2, and p38 and thus impaired cell migration. Moreover, exploiting stress conditions in the absence of plectin, triggers prominent fragmentation of the intermediate filament network (Gregor et al., 2014). In agreement with these findings, cytoplasmic intermediate filaments perceive tension relayed by the upstream mechanosensors and, in response, initiate rearrangements to function as stress buffers. How cytoplasmic intermediate filaments sense tension remains poorly understood. A speculative possibility is that cytoplasmic intermediate filaments alter their conformation or assembly upon stress to reveal cryptic sites crucial for sensing tension. For example, vimentin Cys327 site gets blocked under tension (Johnson et al., 2007; Pérez-Sala et al., 2015). Cytoplasmic intermediate filaments of all types exhibit plasticity in their structural folding which may offer both elasticity and potential for cryptic unmasking. These conformational changes in intermediate filament structure within the polymerized filament could have profound impacts to cell signaling as detailed earlier, and offers a bridge between managing the physical architecture and biochemical signaling.

## Intermediate filaments as determinants of migration

With all of the above in mind, intermediate filaments must be considered as far more than just “intracellular rubberbands.” Attention must be given to intermediate filaments' polymerization state, connections to adhesions, and influence on signal transduction pathways. Like their actin and microtubule counterparts, intermediate filaments have profound influence over cellular functions, with migration being amongst the most dynamic.

A traditional view of intermediate filaments, particularly keratins, is that their association with stable adhesions provides for a general inhibition of migratory potential. And indeed, depletion or mutation of keratin alters, often increasing, migration rates of cancer cells which is likely to contribute to metastasis (Busch et al., 2012), affect invasiveness (Fortier et al., 2013), and wound healing (Morley et al., 2003). Additionally, impaired directional migration has been observed in MCF-7, HeLa, and Panc-1 epithelial cells lacking keratin expression (Long et al., 2006). In contrast, upregulation of vimentin is seen during wound healing (Eckes et al., 1998; Gilles et al., 1999; Rogel et al., 2011; Menko et al., 2014) and carcinoma invasion (Dmello et al., 2016). In addition to being an often used general marker of epithelial-mesenchymal transition (EMT), vimentin has a direct role in the migratory phenotype of cells having undergone EMT (Vuoriluoto et al., 2011; Liu et al., 2015), and declining vimentin levels decrease motility during mesenchymal-epithelial transition (MET; Mendez et al., 2010). Furthermore, treatment of cells with diverse bioactive molecules such as withaferin A (Grin et al., 2012; Menko et al., 2014), acrylamide (Eckert, 1985), okadaic acid (Strnad et al., 2001), orthovanadate (Strnad et al., 2002), or sphingolipids (Beil et al., 2003; Hyder et al., 2015) simultaneously disrupts cytoplasmic intermediate filament arrays into to perinuclear collapse or soluble aggregates and alters the migration rates of cells.

The aforementioned view of keratins as inhibiting migration and vimentin as promoting migration, while convenient, greatly oversimplifies the actual role that intermediate filaments play in migration. In fact, some keratins, such as K14, can promote cell migration, and their expression is correlated with both invasive carcinomas and migration during embryonic development (Sun et al., 2010; Cheung et al., 2013, 2016). Still other keratins, like K19, seem to have multiple functionalities that may greatly depend on the expression levels and more nuanced roles in modification of signal transduction pathways (Ohtsuka et al., 2016; Saha et al., 2017). How then might intermediate filaments impact migration when they are doing more than simply resisting motility?

Different modes of migration, whether random or directed, individual or collective, require the cytoskeleton to generate the structures that drive cell movement. Unique cytoskeletal structures determine and differentiate the protrusive cell front and a retracting rear. Akin to the differences that one sees in actin organization in the front vs. the back (branched actin vs. contractile stress fibers), it has become evident in recent years that cytoplasmic intermediate filaments establish a similarly polarized cytoskeletal network that regulates motility of single migratory cells.

Intermediate filaments extend through the rear and the perinuclear region of the cell, whereas vimentin particles are predominantly present in the lamellipodia (Helfand et al., 2011). Consistent with this correlational observation, it has been shown that increased presence of vimentin particles precedes lamellipodia formation (Helfand et al., 2011). Induced disruption of vimentin intermediate filament networks by microinjection of vimentin mimetic peptide (1A or 2B2) induces membrane ruffling at cell edges previously devoid of lamellipodia (Goldman et al., 1996; Helfand et al., 2011). Furthermore, non-filamentous vimentin or ULF's were shown to be in close proximity with smaller FAs while stable vimentin filaments were in vicinity of large FAs (Terriac et al., 2017). This suggests that assembly states of vimentin seems not only affect lamellipodia formation but may also be involved in establishing the anisotropy of focal contacts and focal adhesions to modulate efficient migration.

Notably, roles for intermediate filaments in migration are not limited to vimentin. In isolated keratinocytes, keratin particles primarily reside in the lamellipodia and keratin intermediate filaments extend through the cell body to the trailing edge (Kolega, 1986; Kolsch et al., 2010). Furthermore, these cells also exhibit asymmetric keratin dynamics, keratin particles prominently forming in the lamellipodia, further grow by elongation and fusion until integration into the peripheral network (Kolsch et al., 2010). In some non-epithelial cells that express keratins such as mesodermal cells, the correlation between cell protrusive polarity and reorganization of keratin intermediate filament network remains (Weber et al., 2012). In single multipolar mesodermal cells, lacking a definite protrusive polarity, keratin intermediate filaments span across the cell cytoplasm and yet are notably absent from protrusions (Weber et al., 2012). It remains to be determined whether the location of the keratin filaments *per-se* is a determinant of protrusive activity. However, in support of this hypothesis computational models predict that lamellipodia formation occurs in the direction opposite to keratin network formation (Kim et al., 2012). Keratins mediate stabilization of hemidesmosomes in some cells, and through promotion of these stable contacts, migration is inhibited (Seltmann et al., 2013b). However, not all cells that express keratins, especially during development while tissues are still establishing themselves, make hemidesmosomes and/or desmosomes. The influence of intermediate filaments on migration may have as much to do with the types of adhesions with which they are associated (focal contacts vs. hemidesmosomes) as it does the intermediate filament subtype expressed (vimentin vs. keratin).

How do intermediate filaments guide migration when cells are moving cohesively? Front-rear polarization depends on mechanical cues exerted at cell-cell junctions. Formation of adherens junctions, but not desmosomes, generates tensile stress in tissues (Harris et al., 2014). Perturbing such intercellular contacts either by function blocking antibodies, chelation of calcium or protein knockdown, attenuates stresses mediated by classical cadherins and collective cell migration (Ganz et al., 2006; Bazellières et al., 2015; Plutoni et al., 2016). Application of local tension on single mesodermal cells induces reorganization of the intermediate filaments at cell-cell adhesion sites via plakoglobin (Weber et al., 2012), coincident with the induction of polarized cell protrusions and directional migratory behavior (Toyoizumi and Takeuchi, 1995; Weber et al., 2012). Similarly, *ex-vivo* embryonic *Xenopus* tissue explants arrange keratin intermediate filaments in a manner similar to single cells under tension (Weber et al., 2012). Reorganization of keratin cytoskeleton is also observed during epithelial sheet migration (Long et al., 2006). Intercellular tissue tension also contributes to integrin mediated traction forces (Dzamba et al., 2009); and conversely, integrin-fibronectin traction forces contribute to tissue tension and affect cell-cell tension by increasing the size of cadherin mediated cell-cell adhesions (Liu et al., 2010; Maruthamuthu et al., 2011). Thus, both cell-cell and cell-ECM interactions establish physicomechanical guidance cues. Extending lamellipodia of repair cells of wound healing are frequently enriched with vimentin particles (Menko et al., 2014). Likewise keratin particles are observed in the leading edge lamellipodia of epithelial cells (Kolsch et al., 2010). Thus, intermediate filaments break the symmetry and are arranged in an asymmetrical array to support polarized migration. We argue that this asymmetry may facilitate establishment of differential front and rear microenvironments necessary for efficient migration.

**Figure 2:**
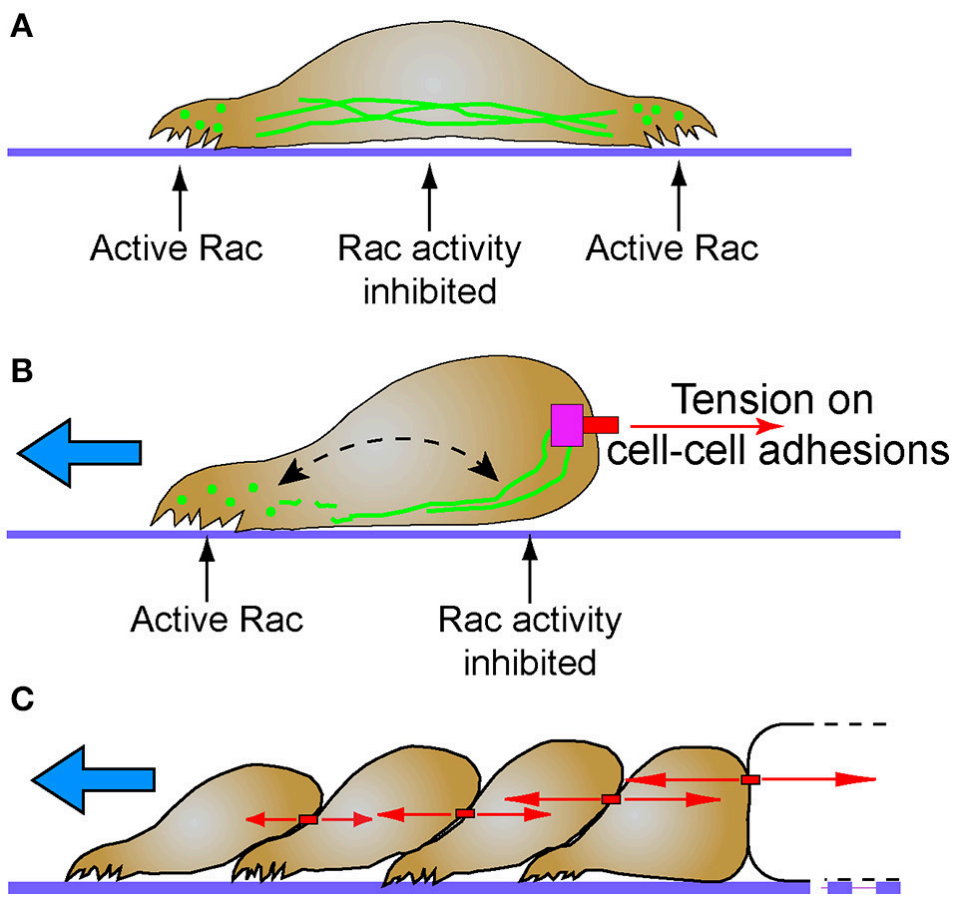
Intermediate filaments and the establishment of cellular subdomains to drive directional migration. **(A)** Intermediate filaments exist in cells as monomer, filament precursors, and mature filaments. While mature intermediate filaments connect to cell adhesions, the nuclear lamina and span across the cell body, they are often notably absent from protrusions. Filament precursors are abundant in protrusions where Rac is active. **(B)** Tension (red arrows) on cell-cell adhesions recruits intermediate filaments. Persistent localization of intermediate filaments proximal to cell-cell adhesions may establish distinct non-protrusive Rac-inhibited zones. Areas of the cell with lesser tension on cell-cell contacts do not recruit intermediate filaments, creating Rac-permissive zones that promote protrusions that lead to directional migration (blue arrow). Despite this subcellular localization, intermediate filaments remain dynamic through non-polar subunit exchange (dashed arrow). **(C)** Stable cell-cell adhesions and the differential intercellular tension present across tissues may promote persistent collective cell migration behavior (blue arrow). Intermediate filaments simultaneously maintain tissue integrity while influencing cell signaling pathways that determine cell polarity and protrusive behavior.

The small GTPase Rac1 is a probable mechanism for the differential localization of cytoplasmic intermediate filaments, and has direct implications to regulation of migration and polarity. Optimum levels of Rac1 play a critical role in protrusion formation to ensure directional cell migration (Pankov et al., 2005). Spatial and temporal activation of Rac1 is sufficient to promote collective cell migration in different models (Theveneau et al., 2010; Wang et al., 2010; Yoo et al., 2010). Additionally, leader cells of the MDCK collectives not only show elevated levels of Rac1, integrin β1, and PI3k, but also inhibition of any of these molecules disrupt the migratory phenotype. Activation of Rac1 which is downstream of integrin β1 and PI3k drives the collective migration (Yamaguchi et al., 2015). In single migratory fibroblasts, local induction of Rac1, promotes disassembly of vimentin intermediate filaments, locally inducing membrane ruffles, while the assembled filaments are maintained in the rear (Helfand et al., 2011). Rac1 activity is negatively regulated by cadherin (Kitt and Nelson, 2011) and plakoglobin (Todorović et al., 2010), proteins which are both intermediate filament interacting molecules. These data suggest that cell-cell contacts may serve a mechanosensing and signaling function by stably recruiting intermediate filaments where they locally suppress Rac activity, and cell protrusions, at the posterior of collectively migrating cells (Figure 2).

The antagonistic relationship between cytoplasmic intermediate filaments and Rac1 may act as a mechanochemical switch that determines which of two mutually exclusive signaling states will occur. A similar switch exists between merlin and Rac1. A negative feedback loop between merlin-Rac1 controls the protrusion promoting state in the front end of the cell and protrusion inhibiting state at the rear end of the cells (Das et al., 2015). Stable cell-cell adhesions promote persistent directionality through this negative feedback loop (Das et al., 2015). Interestingly both intermediate filaments and Merlin are associated with stable cadherin-mediated cell-cell contacts. Perhaps future studies will find a molecular mechanistic link given their common function in regulating polarity of collectively migrating cells.

## Functional roles for intermediate filaments during development

Elucidating the role of many cytoplasmic intermediate filaments in embryonic development has proven to be challenging due to functional redundancy and complexity within the family. Targeted deletion of K18 failed to block embryonic development in mice because of the presence of K19, demonstrating the functional redundancy within the protein family (Magin et al., 1998). However, double K18/K19 null mice display embryonic lethality due to disruption of the extraembryonic trophectoderm (Hesse et al., 2000). Despite this dominant role in the extraembryonic tissue, various keratin knockouts have surprisingly mild developmental phenotypes considering the known roles intermediate filaments play in adhesion. Knockout of all Type II keratins (KtyII^−/−^) still produces an embryo that survives through neurulation and begins organogenesis (Vijayaraj et al., 2009). Nonetheless it should be noted that there is a significant delay in the early development of these KtyII^−/−^ mice up to E8.5 that rapidly attempts to recover to E9.5 but ultimately ends in embryonic lethality (Vijayaraj et al., 2009). Defects in specific tissues at later stages where keratins are expressed argues a role for keratins in late tissue morphogenesis, homeostasis and physiological function (Bouameur and Magin, 2017), but a role for keratins in early embryogenesis has largely remained elusive in mouse models. As with many of the keratin knockout mice, mice lacking vimentin surprisingly undergo embryonic development quite normally, however, exhibit impaired wound healing (Eckes et al., 2000). In some cases, the role of cytoplasmic intermediate filaments only becomes evident upon mechanical and/or chemical stresses (Bouameur and Magin, 2017).

Genetic mouse models have yet to indicate a role for keratins or vimentin in early embryogenesis, but knockout mice have unequivocally revealed a definitive role for keratins in development and maintenance of skin (Bär et al., 2014; Kumar et al., 2015). The epidermal skin is broadly comprised of proliferative basal, stratified suprabasal, and terminally differentiated cornified layers. Each of these layers expresses a unique combinations of Type I and II keratins. Additional keratins, such as K6, K7, K9, K17, K76, have limited expression in the specialized epidermal regions like the palms and hair follicles. In line with prior data that hemidesmosomes and desmosomes provide for mechanical strength of skin, keratin knockout mice have severely fragile skin and the barrier function of the skin is compromised (Bär et al., 2014; Kumar et al., 2015). What is more, perturbation of keratin expression in these layers also results in the disruption of the homeostasis of the epidermis as it matures into distinct layers. Although functional redundancy may obscure the role of specific keratins (Reichelt et al., 2001; Reichelt and Magin, 2002), dysregulation of cell proliferation is a common theme in several keratin knockouts (e.g., K7^−/−^, K8^−/−^, K9^−/−^, K10^−/−^; Bouameur and Magin, 2017). Keratins also coordinate cell growth and protein biosynthesis by accurate localization of GLUT-1 and -3 and consequentially optimize regulation of mTOR pathway as evidenced by keratin Type II knockout mice (Vijayaraj et al., 2009). Collectively, these data point toward an important role for cytoplasmic intermediate filaments in modulating cell growth and proliferation through their impact on cell signaling pathways.

Cells migrate collectively in a coordinated manner to accomplish various tasks for development of the organism, from gametogenesis to morphogenesis to organogenesis. Collective cell migration allows whole groups of cells to move toward their final destination most efficiently while maintaining tissue cohesivity and tissue-specific characteristics. All the while, these cells can transmit signals to each other and effectively navigate the complex and changing environment within the developing embryo.

Disruption of the keratin network in the amphibian embryo tells quite a different story than mice about the importance of intermediate filaments in early embryogenesis. Disruption of keratin by either targeting protein expression (Heasman et al., 1992; Weber et al., 2012) or filament assembly (Klymkowsky et al., 1992) impairs mesodermal involution and blastopore closure during gastrulation. Pointing to a role in collective migration events, polarized protrusive cell behavior of the mesoderm is lost in the absence of K8 expression in *Xenopus* embryos (Weber et al., 2012).

Collective cell movements are also perturbed in keratin mutant mice, albeit at stages of organogenesis and tissue maintenance. Vimentin plays a role in promoting stemness of mammary epithelial cells which provide the basis for mammary gland growth. Ductal outgrowth is significantly delayed in mammary glands from vimentin knockout mice and the lumen is slightly enlarged (Virtakoivu et al., 2017). Vimentin expression in stromal and basal epithelial layers is accompanied by expression of keratins in the basal (K14) and luminal (K8/18) layers of the mammary epithelia (Sun et al., 2010; Virtakoivu et al., 2017). Both populations of mammary cells are involved in the branching morphogenesis of the tissue. Live cell imaging studies have shown that K8/18+ mammary epithelial cells collectively migrate during this process (Ewald et al., 2008) and other studies have indicated a pro-migratory role of K14 expressing mammary cells in collective invasion at the epithelial-stromal boundary (Cheung et al., 2013). Interestingly during the initial development of the mammary placode in the embryonic mouse, these invasive migratory cells express both K8 and K14 (Sun et al., 2010). Only recently have selective promoters for basal mammary epithelial cells become available. It will be interesting to determine whether knockout of K14 and/or K8 functionally inhibits mammary development.

Morphogenesis of epidermal and muscle tissue in *Caenorhabditis elegans* provides a particular elegant example of the interplay between intermediate filaments and mechanotransduction pathways during development. Muscle-generated tension within the epidermis induces recruitment of the adaptor protein GIT-1 and its partner PIX-1, a Rac GEF, to hemidesmosomes (Zhang et al., 2011). With PIX-1 at the hemidesmosome, Rac is activated, which further stimulates PAK-1 activity and subsequent phosphorylation of intermediate filaments (Zhang et al., 2011). Phosphorylation of intermediate filaments through this mechanism drives remodeling and maturation of the hemidesmosome and the associated intermediate filament network (Zhang et al., 2011). Hemidesmosomes behave as mechanosensors that further relay the tension by activation of specific signaling pathway that promotes epithelial morphogenesis (Zhang et al., 2011). Indeed, coordination between the epidermis and muscle cells is absolutely essential to epidermal morphogenesis that elongates the worm, and cytoplasmic intermediate filaments are vital to this process (Woo et al., 2004).

Migration driven by cell-cell adhesions has roles very early in development, even as early as development of gametes. Tension sensing through E-cadherin plays a critical role in controlling directionality of migration of border cells in the Drosophila ovary (Cai et al., 2014). As with many collectively migrating cells, asymmetric Rac activity also plays a key role in the steering of these migrating collectives (Wang et al., 2010; Yoo et al., 2010). For some time, cytoplasmic intermediate filaments were believed to be absent from many non-chordates including arthropods. Recently however, it was found that the tropomyosin-1 gene (Tm1) produces a unique isoform, Tm1-I/C, which has many cytoplasmic intermediate filament-like characteristics, including the tripartite head-rod-tail structure and the ability to anneal end-to-end and spontaneously polymerize into filaments of similar diameter to intermediate filaments (Cho et al., 2016). Knockdown of this Tm1 isoform impairs border cell migration, unlike knockdown of other Tm1 isoforms. Moreover, actin stress fiber organization is perturbed in cells lacking Tm1-I/C, despite that Tm1-I/C does not co-localize with actin (Cho et al., 2016). While the inhibition of border cell migration in Tm1-I/C knockdown cells could be entirely structurally related to facilitating adhesions and/or enabling protrusions, it certainly is appealing to speculate that Tm1-I/C, like keratin and vimentin intermediate filaments, tethers to cell adhesions and allows for mechanotransduction between these adhesions and signaling mechanisms regulating cell protrusive behavior.

## What is next for intermediate filaments?

Intermediate filaments are the next frontier for understanding how cells cope with mechanical stimuli and integrate these signals with cellular function. Current data bolsters the notion that cytoplasmic intermediate filaments provide a unique scaffolding framework that regulates major mechanotransduction events initiated through cellular adhesions. Moreover, intermediate filaments function in these mechanotransduction processes in a non-redundant manner that cannot be compensated by other cytoskeletal networks during development.

New innovative methods will have to be devised to tackle the details of how intermediate filaments are regulated in terms of turnover, dynamic exchange, and other remodeling events *in vivo*. Although, various signaling pathway relationships to intermediate filaments have been found, the molecular mechanism by which intermediate filaments effect signaling is not always clear. For a few proteins, direct interaction with intermediate filaments are known to exist. The next step will be to determine how strain on intermediate filaments impacts these binding partners and their activity and/or intermediate filament polymerization state. Subcellular compartmentalization of the different intermediate filament polymerization states (i.e., intermediate filament proteins as filaments or particles) is likely to have important consequences to the local cellular activity. Intermediate filament filaments and particles may form a differential composite network that, in coordination with the other cytoskeletal elements, endures and responds to changing physical parameters that cells experience during development.

Significant headway has been made to investigate the relationship between intermediate filaments and the actin and microtubule networks. Still, questions remain particularly related to the *in vivo* functionality of intermediate filament elasticity. If intermediate filaments cannot independently generate force because of the lack of associated motor proteins, is it possible for them to be pre-stressed by nearby actomyosin or microtubule-kinesin/dynein networks? In this regard, intermediate filaments could store substantial potential energy and enable cell contractility through a non-actomyosin mechanism. Both actomyosin and intermediate filaments could work cooperatively as dynamic elastic components. Likewise, nuanced differences between various keratin proteins and vimentin in regulation, turnover rates, and mechanical properties are likely optimized for different cell and tissue-specific functions.

Intermediate filaments as elastic, but resilient, cytoskeletal structures may undergo conformational changes due to mechanical stresses that unmask cryptic binding sites within the polymerized filament. If great enough, these stresses might otherwise rupture a cell or another cytoskeletal component, but intermediate filaments are uniquely suited to cope with these greater forces. For cytoplasmic intermediate filaments, they become a strain sensor- in essence, only permitting certain cell signaling events to occur when strain is applied. Developmental morphogenesis and cell migration are but two processes for which these signals would be important, yet important ones since dramatic tissue shaping occurs on a rapid timescale. As much as intermediate filaments have historically been touted as the keepers of cellular mechanical integrity, investigating them as dynamic components of cellular mechanosensor complexes is demanded.

If we think about different types of adhesions as an interdependent network, then perhaps we can confer a similar thought process to how we think about cytoskeletal networks. Given the coordination in localization, function, and integration with signal transduction pathways, our longstanding conceptual models of discrete adhesive structures with separate cytoskeletal networks may be long overdue for a re-thinking. As much as we have learned about the role of cell adhesions and actomyosin in force-induced signal transduction in the last decade, the potential exists for an equally robust phase of discovery about intermediate filaments and their role in mechanotransduction during development.

## Author contributions

RS and GW shared equally in the conceptual development, literature research, and writing of the manuscript.

### Conflict of interest statement

The authors declare that the research was conducted in the absence of any commercial or financial relationships that could be construed as a potential conflict of interest.
